# Hepatitis B and C virus infection among healthcare workers in Africa: a systematic review and meta-analysis

**DOI:** 10.1186/s12199-021-00983-9

**Published:** 2021-06-02

**Authors:** Daniel Atlaw, Biniyam Sahiledengle, Zerihun Tariku

**Affiliations:** 1 Department of Human Anatomy, School of Medicine, Goba Referral Hospital, Madda Walabu University, Goba, Ethiopia; 2 Department of Public Health, School of Health Science, Goba Referral Hospital, Madda Walabu University, Goba, Ethiopia; 3 Department of Public Health, College of Medicine and Health Sciences, Dire Dawa University, Dire Dawa, Ethiopia

**Keywords:** Hepatitis B, Hepatitis C, Health care workers, Africa

## Abstract

**Background:**

Healthcare workers are at risk of acquiring hepatitis B and C virus infections through patients’ blood and bodily fluids exposure. So far, there is no pooled data that shows the prevalence of HBV and HCV among health care workers in Africa. This study aimed to determine the pooled prevalence of hepatitis B and C infections among health care workers in Africa.

**Methods:**

Studies reporting the prevalence of HBV and HCV were identified from major databases and gray literature. PubMed, CINAHL, POPLINE, ScienceDirect, African Journals Online (AJOL), and Google Scholar were systematically searched to identify relevant studies. A random-effect model was used to estimate the pooled prevalence of hepatitis B and C among health care workers in Africa. The heterogeneity of studies was assessed using Cochran Q statistics and I^2^ tests. Publication bias was assessed using Begg’s tests.

**Result:**

In total, 1885 articles were retrieved, and 44 studies met the inclusion criteria and included in the final analysis. A total of 17,510 healthcare workers were included. The pooled prevalence of hepatitis B virus infection among health care workers in Africa is estimated to be 6.81% (95% CI 5.67–7.95) with a significant level of heterogeneity (I^2^ = 91.6%; *p* < 0.001). While the pooled prevalence of hepatitis C virus infection using the random-effects model was 5.58% (95% CI 3.55–7.61) with a significant level of heterogeneity (I^2^ = 95.1%; *p* < 0.001).

**Conclusion:**

Overall, one in fifteen and more than one in twenty healthcare workers were infected by HBV and HCV, respectively. The high burden of HBV and HCV infections remains a significant problem among healthcare workers in Africa.

**Supplementary Information:**

The online version contains supplementary material available at 10.1186/s12199-021-00983-9.

## Background

Hepatitis B virus (HBV) is a DNA virus and hepatitis C virus ( HCV) is an RNA virus [1]. Both HBV and HCV are transmitted by parenteral or mucosal exposure to infected blood and body fluids [1, 2]. Hepatitis B and C viruses are the most common causes of chronic hepatitis, cirrhosis, and hepatocellular carcinoma resulting in high morbidity and mortality all over the world [3, 4]. Hepatitis B virus is more contagious [2] and HCV is a predominant cause of chronic hepatitis [5].

About 350 million people are chronically infected with HBV [6], and 150 million people have chronic hepatitis C virus infection [7]. According to the World Health Organization (WHO), about 14 million people are chronically infected with hepatitis B, and nine million people are chronically infected with hepatitis C in the European region in 2011 [8]. The majority of HBV and HCV infected cases are living in developing countries of sub-Saharan Africa [6]. Globally, HBV and HCV together accounted for an estimated 1.34 million deaths in the year 2015 [9] and in 2013 viral hepatitis infection was the seventh foremost cause of global mortality [10].

The transmission risk of viral hepatitis among health care workers (HCWs) is of great concern. The risk of acquiring hepatitis B virus by HCWs is four times greater than that of the general population [11, 12]. Healthcare workers are usually infected by HBV and HCV via occupational exposure to blood and bodily fluids [11, 13–15]. The circumstance is awful in Africa and Asia where 90% of worldwide hepatitis infections occur [16, 17]. In developing countries, 40–60% of HBV infections in HCWs were attributed to professional hazards [18]. Health care workers are vulnerable to contaminated sharp injuries which constitute a major source of hepatitis B infection, with an estimated 66,000 cases and 261 deaths annually in developing countries [19, 20]. Further, about half of African HCWs are occupationally exposed to blood and body fluids [21, 22]. The evidence available suggests that many HCWs in Africa are at higher risk of hepatitis B infection [23].

Understanding the relative contribution of HBV and HCV to liver disease burden is important for setting public health priorities and guiding prevention programs [24]. In spite of recommendations on hepatitis B vaccination, the immunization rates among health professionals have remained consistently low in African countries [3]. A meta-analysis conducted in 2018 also reported that only a quarter of African HCWs were fully vaccinated against hepatitis B virus [23].

So far, different studies have been conducted in Africa about the prevalence of HBV and HCV infections among HCWs, but the finding was inconsistent on HBV [25–57] and HVC [58–73], and the pooled prevalence is still uncertain. For instance, the prevalence of HBV was 1.4% in Egypt [33], 2.4% in Ethiopia [29], 17.8% in Senegal [54], and 25.7% in Nigeria [55], while the prevalence of HCV was 0.4% in Ethiopia [60], 1.3 in Rwanda [65], and 16.7% in Egypt [58]. Up to date, no pooled data estimate shows the prevalence of HBV and HCV among HCWs in Africa. Therefore, this study aimed to determine the pooled prevalence of hepatitis B and C infections among HCWs in Africa.

## Methods

### Study design and reporting

The protocol of this study was registered in the International Prospective Register of Systematic Reviews (PROSPERO), the University of York Centre for Reviews and Dissemination (ID number CRD42021230905). A systematic review and meta-analysis were conducted to estimate the pooled prevalence of HBV and HCV among HCWs in Africa. This review and meta-analysis were conducted according to the guideline of Preferred Reporting Items for Systematic reviews and Meta-Analysis (PRISMA) (Supplementary file 1).

### Eligibility criteria

Studies conducted in Africa that have reported the prevalence of hepatitis B or/and hepatitis C and fulfilled the following criteria were included.

- *Population*. Healthcare works (HCWs) with direct contact to patients
- *Study designs*. Observational studies reporting the prevalence of hepatitis B or/and hepatitis C were eligible for this systematic review and meta-analysis
- *Language*. Articles published in English were considered.
- *Publication status*. Both published and unpublished articles were considered.
- *Year of publication*. All publications reported up to December 31, 2020, were considered.
- *Exclusion criteria*. Studies that reported hepatitis B prevalence but do not have a separate outcome for HBsAg

### Operational definition

Healthcare workers are referred to as full-time employees working in a healthcare setting whose activities involve direct contact with patients. Hence, we incorporated studies, which have involved physicians, nurses, and laboratory technicians mainly.

#### Outcome of interests and measurement

Prevalence hepatitis B and C virus infection were the outcome of interests. The prevalence of HBV was calculated from primary studies by dividing the number of HCWs tested for HBsAg and reported as positive to the total number of health care workers multiplied by 100. Similarly, the prevalence of hepatitis C virus infection was calculated by dividing the number of HCWs tested for serum HCV antibody, and reported positive to the total number of health care workers multiplied by 100.

### Search strategy

A systematic search of works of literature was conducted by the authors to identify all relevant primary studies. Both published and unpublished articles on the prevalence of hepatitis B virus and hepatitis C virus infections in Africa were identified through a literature search. The databases used to search for studies were PubMed, Science Direct, CINAHL, Popline, Cochrane Library, and African Journals Online (AJOL) and gray literature was searched on Google and Google Scholar until December 31, 2020. The following key search terms and Medical Subject Headings [MeSH] were used “prevalence” OR “magnitude” AND “hepatitis B” OR “hepatitis C” AND “health care workers” OR “health professionals” AND “Africa [MeSH]” were used separately or in combination with the Boolean operator’s terms “AND” and “OR” (Supplementary file 2). In addition, the reference lists of the retrieved studies were also scanned to access additional articles and screened against our eligibility criteria.

#### Data Extraction

In this review, all the searched articles were exported into the EndNote version X8 software, and subsequently, the duplicate articles were removed. Screening of retrieved articles titles, abstracts, and full-text quality were conducted independently by two review authors (DA and BS) based on the eligibility criteria. Any disagreement between the two review authors was resolved by consensus through discussion. Afterward, full-text articles were retrieved and appraised to approve eligibility. Finally, the screened articles were compiled together by the two investigators. Data were extracted using a data extraction format in Microsoft Office Excel software. The data extraction tool consists of the name of the author(s), year of publication, country and sub-region, study design, sample size, and prevalence of hepatitis B and hepatitis C (Table 1).

**Table 1:** Characteristics of included studies in meta-analysis on prevalence of hepatitis B and C in Africa

Author name	Year of publication	Country	Study design	Sample size	Prevalence of HBV	Prevalence of HCV	Prevalence of HBV in nurses	Prevalence of HBV in laboratory technician	Prevalence of HBV in physician
Desalegn et al. [27]	2013	Ethiopia	Cross-sectional	254	2.4		4		3.8
Ziraba et al. [53]	2010	Uganda	Cross-sectional	370	8.1		8.61	18.18	3.8
Mueller et al. [51]	2016	Tanzania	Cross-sectional	598	7				
Nail et al. [50]	2008	Sudan	Cross-sectional	211	2.3		3.1		
Abiola et al. [25]	2016	Nigeria	Cross-sectional	134	1.5		1.15		2.23
Abdelwahab et al. [58]	2012	Egypt	Cross-sectional	842	1.5	16.7			
Braka et al. [28]	2006	Uganda	Cross-sectional	311	9		10.58	11.11	2.44
Djeriri et al. [31]	2008	Morocco	Cross-sectional	285	5		1.5		1.85
Ngekeng et al. [48]	2018	Cameroon	Cross-sectional	281	5				
Elmaghloub et al. [33]	2017	Egypt	Cross-sectional	564	1.4				
Elmukashfi et al. [34]	2012	Sudan	Cross-sectional	843	6				
Elduma and Saeed [36]	2006	Sudan	Cross-sectional	245	4.9				
Fritzsche et al. [59]	2015	Cameroon	Cross-sectional	237	6.3	1.7	7.29	2.7	6.25
Gebremariam et al. [38]	2018	Ethiopia	Cross-sectional	332	4.52		4.3	4.44	5
Hebo et al. [60]	2019	Ethiopia	Cross-sectional	240	2.5	0.4			
Mafopa et al. [62]	2019	Sierra Leone	Cross-sectional	81	4.9	2.5			
Alese et al. [26]	2016	Nigeria	Cross-sectional	187	1.1				
Munier et al. [63]	2013	Egypt	Cohort	597	7.3	7.2			
Kisangau et al. [57]	2018	Kenya	Cross-sectional	295	4.5				
Jean-Baptiste et al. [61]	2018	Ivory cost	Cross-sectional	632	8.4	1.4	19.48	28	38.14
Souly et al. [64]	2016	Moroccoo	Cross-sectional	1189	3.2	1.3	4.03	3.45	2.7
Orji et al. [47]	2020	Nigeria	Cross-sectional	236	2.1				
Yizengaw et al. [43]	2018	Ethiopia	Cross-sectional	268	2.6		1.87	6.45	
Ndako et al. [49]	2014	Nigeria	Cross-sectional	188	17		13.4	12.9	21.43
Elikwu et al. [32]	2016	Nigeria	Cross-sectional	100	7		7.02		5.88
Geberemicheal et al. [37]	2013	Ethiopia	Cross-sectional	110	7.3				
Shao et al. [46]	2018	Tanzania	Cross-sectional	442	5.7		3.7	10.81	5.38
Sondlane et al. [45]	2016	South Africa	Cross-sectional	314	2.9				
Tatsilong et al. [44]	2016	Cameroon	Cross-sectional	100	11		10.2	25	
Kateera et al. [65]	2014	Rwanda	Cross-sectional	378	2.9	1.3			
Elbahrawy et al. [66]	2017	Egypt	Cross-sectional	564		8.7			
Akazong et al. [27]	2020	Cameroon	Cross-sectional	338	10.6		12.5	8.89	5.88
Amiwero et al. [67]	2017	Nigeria	Cross-sectional	248	11.3	2.4	13.04		11.76
Daw et al. [30]	2000	Libya	Cross-sectional	459	4				
Romieu et al. [54]	1989	Senegal	Cross-sectional	775	17.8				
Qin et al. [52]	2018	Sierra Leone	Cross-sectional	211	10				
Elzouki et al. [35]	2014	Libya	Cohort	601	1.8		2.41	0.91	0.74
Ndongo et al. [56]	2016	Cameroon	Cross-sectional	1790	8.7				
Vardas et al. [68]	2002	South Africa	Cross-sectional	399		1.8			
Lungosi et al. [41]	2018	DR Congo	Cross-sectional	97	18.6				
Massaquoi et al. [39]	2018	Sierra Leone	Cross-sectional	447	8.7				
Mbaawuaga et al. [40]	2019	Nigeria	Cross-sectional	221	10.6		11.63		
Sani et al. [69]	2011	Nigeria	Cross-sectional	100	19	5			
Zayet et al. [72]	2015	Egypt	Cross-sectional	215	3.1	5.2	32		14.29
Kefenie et al. [42]	1989	Ethiopia	Cross-sectional	432	9.2		8.82	6.45	
El-Sokkary et al. [70]	2017	Egypt	Cross-sectional	69		40.6			
Belo et al. [55]	2000	Nigeria	Cross-sectional	167	25.7				
Gyang et al. [73]	2016	Nigeria	Cross-sectional	155	8.5	6.5	2.74		3.13

### Risk of bias assessment

The qualities of the included studies were assessed and the risks for biases were refereed using the Joanna Briggs Institute (JBI) quality assessment tool for the prevalence studies [74]. Two reviewers (DA and BS) assess the quality of included studies independently and the discrepancy between the two review authors was resolved by reaching a consensus through discussion. The assessment tool consists of nine parameters: (1) appropriate sampling frame, (2) proper sampling technique, (3) adequate sample size, (4) study subject and setting description, (5) sufficient data analysis, (6) use of valid methods for the identified conditions, (7) valid measurement for all participants, (8) using appropriate statistical analysis, and (9) adequate response rate [74]. Failure to satisfy each parameter was scored as 1 if not 0. When the information provided was not adequate to assist in making a judgment for a specific item, we agreed to grade that item as 1 (failure to satisfy a specific item). The risks for biases were classified as either low (total score, 0 to 2), moderate (total score, 3 or 4), or high (total score, 5 to 9) (Table 2).

**Table 2:** Risk bias assessment of individual studies included for meta-analysis on prevalence of hepatitis B and C in Africa

Wow	Year of publication	Q1	Q2	Q3	Q4	Q5	Q6	Q7	Q8	Q9	Total score	Risk of bias
Desalegn et al. [29]	2013	1	0	0	0	0	1	1	0	0	3	Moderate
Ziraba et al. [53]	2010	0	0	0	0	0	0	0	0	0	0	Low
Mueller et al. [51]	2016	0	0	0	0	0	1	0	0	0	1	Low
Nail et al. [50]	2008	1	1	0	1	0	0	0	1	1	5	High
Abdelwahab et al. [58]	2012	1	0	1	0	0	1	1	1	0	5	High
Braka et al. [28]	2006	0	1	0	0	0	0	0	1	0	2	Low
Djeriri et al. [31]	2008	1	0	0	0	0	0	0	0	1	2	Low
Ngekeng et al. [48]	2018	0	0	0	0	1	1	0	1	1	4	Moderate
Elmaghloub et al. [33]	2017	1	0	0	1	0	0	0	0	1	3	Moderate
Elmukashfi et al. [34]	2012	0	1	0	1	0	0	0	1	1	4	Moderate
Elduma and Saeed [36]	2006	1	0	0	1	0	0	0	1	1	4	Moderate
Fritzsche et al. [59]	2015	1	0	0	1	0	1	1	0	1	5	High
Gebremariam et al. [38]	2018	0	0	0	0	0	1	0	0	0	1	Low
Munier et al. [63]	2013	1	0	0	1	0	0	0	0	1	3	Moderate
Kisangau et al. [57]	2018	0	0	0	0	1	0	0	1	0	2	Low
Jean-Baptiste et al. [61]	2018	1	0	0	1	0	1	0	0	1	4	Moderate
Souly et al. [64]	2016	1	0	0	1	0	0	0	0	1	3	Moderate
Orji et al. [47]	2020	0	0	0	0	0	0	0	1	0	1	Low
Yizengaw et al. [43]	2018	0	0	0	0	0	0	0	0	0	0	Low
Ndako et al. [49]	2014	0	0	0	1	0	1	1	0	1	4	Moderate
Elikwu et al. [32]	2016	1	0	0	1	0	0	0	1	0	3	Moderate
Geberemicheal et al. [37]	2013	1	0	0	1	0	0	0	0	0	2	Low
Shao et al. [46]	2018	0	0	0	0	1	1	0	0	0	2	Low
Sondlane et al. [45]	2016	0	0	0	0	0	0	1	0	0	1	Low
Tatsilong et al. [44]	2016	0	0	0	0	0	1	0	1	0	2	Low
Kateera et al. [65]	2014	0	0	0	0	1	0	0	1	0	2	Low
Elbahrawy et al. [66]	2017	1	0	0	1	0	0	0	0	0	2	Low
Akazong et al. [27]	2020	0	0	0	0	0	0	1	0	0	1	Low
Amiwero et al. [67]	2017	0	0	1	0	0	0	1	1	0	3	Low
Daw et al. [30]	2000	0	0	1	0	1	0	1	1	0	4	Moderate
Romieu et al. [54]	1989	1	0	0	1	0	1	1	1	1	6	High
Qin et al. [52]	2018	0	0	0	0	0	1	0	0	0	1	Low
Elzouki et al. [35]	2014	0	0	0	0	1	0	0	0	0	1	Low
Ndongo et al. [56]	2016	0	1	0	0	0	0	0	0	0	1	Low
Vardas et al. [68]	2002	0	1	0	0	1	1	1	0	0	4	Moderate
Lungosi et al. [41]	2018	0	0	0	0	1	0	1	0	0	2	Low
Massaquoi et al. [39]	2018	0	0	0	0	0	0	0	1	0	1	Low
Mbaawuaga et al. [40]	2019	1	0	0	0	0	0	0	1	0	2	Low
Sani et al. [69]	2011	1	1	1	0	1	0	0	1	0	5	High
Zayet et al. [72]	2015	0	1	1	0	1	0	1	1	1	6	High
Kefenie et al. [42]	1989	1	0	0	1	0	0	0	0	0	2	Low
El-Sokkary et al. [70]	2017	0	0	0	0	0	1	0	0	0	1	Low
Belo et al. [55]	2000	1	1	1	0	0	0	0	0	1	4	Moderate
Gyang et al. [73]	2017	0	0	0	1	0	0	0	0	0	1	Low

The risk of bias was classified as either low (total score, 0 to 2), moderate (total score, 3 or 4), or high (total score, 5 to 9)

Q1 = Was the sample frame appropriate to address the target population?

Q2 = Were study participants sampled in an appropriate way?

Q3 = Was the sample size adequate?

Q4 = Were the study subjects and the setting described in detail?

Q5 = Was the data analysis conducted with sufficient coverage of the identified sample?

Q6 = Were valid methods used for the identification of the condition?

Q7 = Was the condition measured in a standard, reliable way for all participants?

Q8 = Was there appropriate statistical analysis?

Q9 = Was the response rate adequate, and if not, was the low response rate managed appropriately?

### Statistical methods and analysis

The extracted data were imported into STATA version 14 software for statistical analysis. The heterogeneity among all included studies was assessed by I^2^ statistics and Cochran Q test. In this meta-analysis, the tests indicate that the presence of significant heterogeneity among included studies (I^2^ = 91.6, *P*-value < 0.001). Thus, a random-effects model was used to analyze the data. Pooled prevalence along their corresponding 95% CI was presented using a forest plot. Sub-group analyses for the prevalence of hepatitis B and C were performed by sub-regions of Africa, sample size, year of publication, and professions of HCWs.

### Publication bias

In this meta-analysis, the presence of publication bias was evaluated using funnel plots and Begg’s tests at a significance level of less than 0.05.

### Sensitivity analysis

To identify the source of heterogeneity, a leave-one-out sensitivity analysis was employed.

## Results

### Description of included studies

**Fig. 1:**
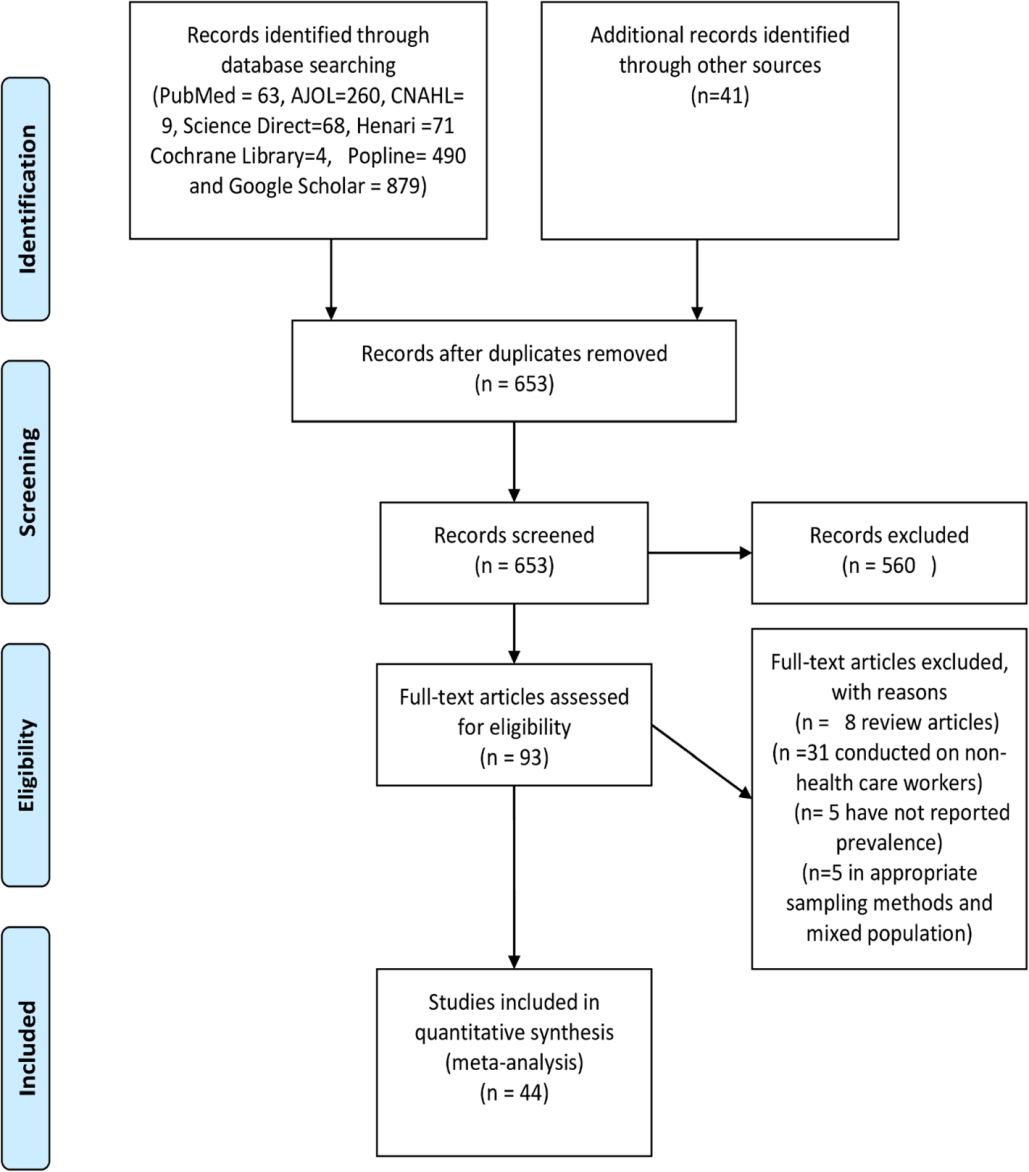
Flow diagram of systemic review and meta-analysis on prevalence of hepatitis B and C among health care workers in Africa, 1989–2021

About 1885 studies were retrieved from initial electronic searches using international databases and google search. The database included PubMed (*n* = 63), ScienceDirect (*n* = 68), Hinari (*n* = 71), Google Scholar (*n* = 879), Cochrane Library (*n* = 4), AJOL (*n* = 260), CINAHL (*n* = 9), POPLINE (*n* = 490), and the remaining (*n* = 41) studies were identified through manual search. Of these, 1332 duplicates were removed, the remaining 553 articles were screened by title and abstract, and 460 articles were excluded after reading their titles and abstracts. Ninety-three full-text articles remained and were further assessed for their eligibility. Finally, based on the pre-defined inclusion and exclusion criteria, a total of 44 articles were included in the meta-analysis and data were extracted for the final analysis (Fig. 1).

### Characteristics of the included studies

Of 44 articles included in this review and meta-analyses, 5 were conducted in Ethiopia, 2 in Uganda, 2 in Tanzania, 3 in Sudan, 8 in Nigeria, 6 in Egypt, 5 in Cameron, 2 in Morocco, 2 in Sierra Leone, 2 in Rwanda, 1 in Kenya, 1 in Côte d’Ivoire, 2 in south Africa, 1 in Senegal, 1 in DR Congo, and 2 in Libya (Table 1).

A total number of 17,510 HCWs participated in this study. The lowest sample size was reported from Egypt (n = 69) and the highest was from Cameron (*n* = 1790). Among the included studies, 31 of them reported the prevalence of HBV, 3 of them presented the prevalence of HCV, and 10 studies reported the prevalence of both HBV and HCV (Table 1).

The latest article was published in 2020, and the earliest study was concluded in 1989. The prevalence of hepatitis B among African HCWs ranged from 1.4% in Egypt to 25.7% in Nigeria. The prevalence of hepatitis C varied from 0.4% in Ethiopia to 40.6% in Egypt (Table 1).

### Socioeconomic status of African countries included in this meta-analysis

Out of sixteen countries included in this meta-analysis, 6 (37.50%) of them have gross national income (GNI) per capita less than $1036, 8 (50%) of them have GNI per capita between $1036 to 4036, and 2 (12.50%) of them have GNI per capita above $4036. In terms of governmental health care expenditure, about 9 (56.25%) of included countries have less than 30% health expenditure from domestic government funding and 2 (12.50%) have higher than 50% of health expenditure from domestic government funding. In addition, out of the sixteen countries, 7 (43.75%) of them have universal health coverage less than 50% and one country has universal health coverage greater than 60% (Table 3).

**Table 3:** Socioeconomic characteristics of African countries included in meta-analysis for prevalence of HBV and HCV in Africa

Countries	GNI per capita (US$) (word bank.org 2019)	Governmental health care expenditure (%) (africanhealthcarestats.org)	Universal health coverage (%) (healthdata.org)	Classification by World Bank (world bank data.org 2020)
Nigeria	2030	13	38.3	Low-middle income
Ethiopia	850	28	46.5	Low income
Sudan	590	19	51.8	Low income
Egypt	2690	29	54.8	Low-middle income
DR Congo	530	12	45.2	Low income
Sierra Leone	540	11	42.1	Low income
Libya	7640	63	66.3	Upper-middle income
Cameron	1500	13	42.3	Low-middle income
Senegal	1460	33	49.6	Low-middle income
Rwanda	830	34	59.4	Low income
South Africa	6040	54	59.7	Upper-middle income
Kenya	1750	36	51.6	Low-middle income
Cote d’Ivoire	2290	26	43.0	Low-middle income
Tanzania	1080	41	55.2	Low-middle income
Uganda	780	17	52.7	Low income
Morocco	3190	47	58.0	Low-middle income

### Prevalence of hepatitis B and C infection among health HCWs in Africa

**Fig. 2:**
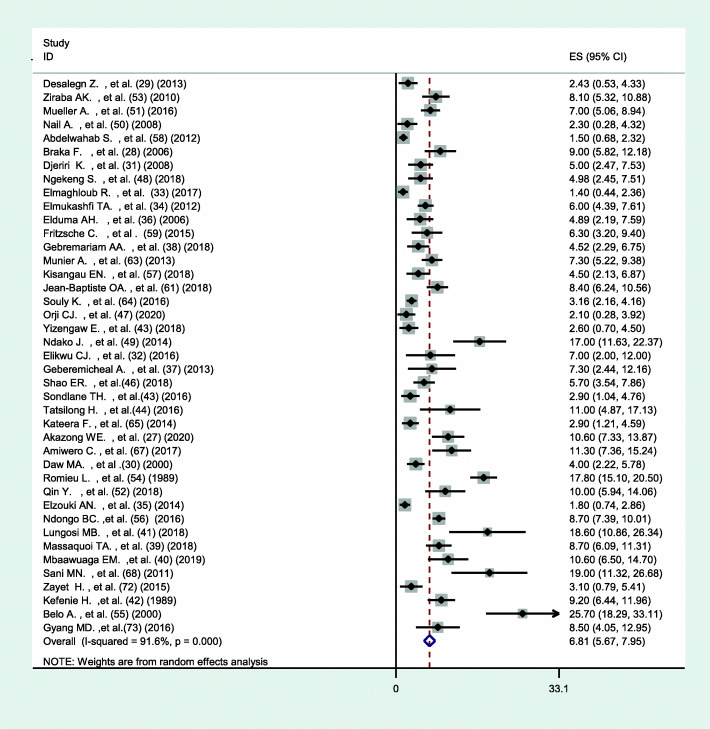
Forest plot showing the pooled prevalence of hepatitis B among HCWs in Africa

**Fig. 3:**
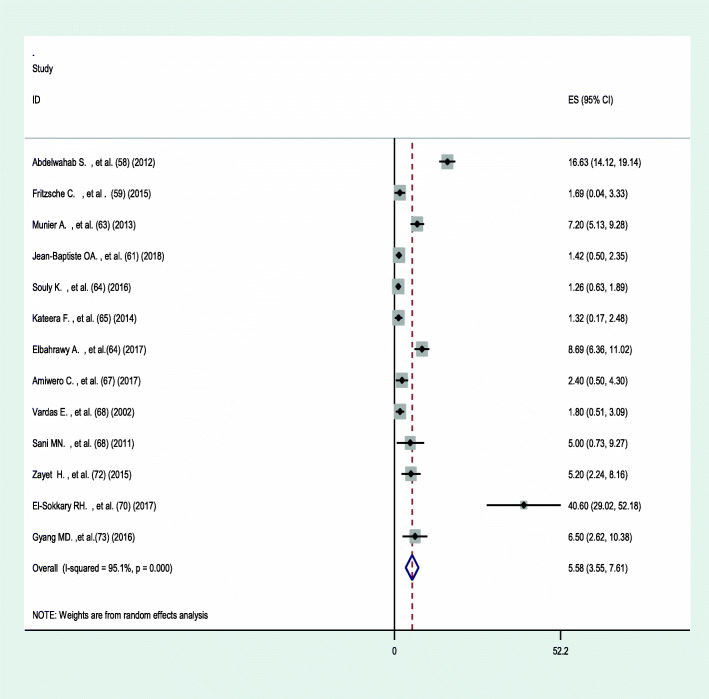
Forest plot showing the pooled prevalence of hepatitis C among HCWs in Africa

The pooled prevalence of hepatitis B among HCWs in Africa using the random-effect model was estimated to be 6.81% (95% CI 5.67–7.92) with a significant level of heterogeneity (I^2^ = 91.6%; *p* < 0.001) (Fig. 2). While the pooled prevalence of hepatitis C using the random-effects model was 5.58% (95% CI 3.55–7.61) with a significant level of heterogeneity (I^2^ = 95.1%; *p* < 0.001) (Fig. 3).

### Sub-group analysis

To identify the possible source of heterogeneity, sub-group analysis was conducted by sub-regions of Africa, sample size, year of publication, and professions of HCWs.

The prevalence of hepatitis B was found to be highest in Western Africa 11.67% (95% CI 8.21–15.17), and the lowest was reported from Northern Africa 3.50% (95% CI 2.41–4.58). Heterogeneity has been shown to vary from I^2^ = 72 to 91.6% on this estimate (Table 4). This meta-analysis also found that the prevalence of hepatitis C infection varied between different sub-regions of Africa, and the highest prevalence was found in Northern Africa, 11.23% (95% CI 5.45–17.01), with significant heterogeneity I^2^ = 97.8% and the lowest prevalence was identified in Eastern Africa 1.32% (95% CI 0.16–2.47) (Table 5).

**Table 4:** Showing sub-group analysis of HBV prevalence by sample size, year of publication, regions of Africa, and HCWs professions in Africa

	Prevalence of HBsAg	95% Confidence interval	Heterogeneity (I^2^%)	*P*-value
Sub-group analysis by sample size				
1. < 101	11.33	6.17–16.50	76.0	*P* = 0.002
2. 101–384	5.57	4.31–6.83	85.7	*P* < 0.001
3. 385–1000	6.42	4.24–8.61	95.1	*P* < 0.001
4. > 1000	5.91	0.48–11.54	97.6	*P* < 0.001
Sub-group analysis by year of publication				
1. < 2001	12.32	3.32–21.39	97.1	*P* < 0.001
2. 2001–2010	5.79	3.43–8.16	88.3	*P* < 0.001
3. 2011–2021	5.71	4.68–6.74	87.7	*P* < 0.001
Sub-group analysis by regions of Africa				
1. North Africa	3.50	2.41–4.58	83.8	*P* < 0.001
2. East Africa	5.51	4.03–6.99	77.5	*P* < 0.001
3. Middle Africa	8.77	6.32–11.21	72.2	*P* = 0.003
4. Western Africa	11.69	8.21–15.17	91.8	*P* < 0.001
5. Southern Africa	2.90	1.04–4.99	-	-
Sub-group analysis by professions				
1. Physician	6.30	3.54–9.07	81.8	*P* < 0.001
2. Nurses	6.31	4.23–8.40	84.5	*P* < 0.001
3. Laboratory staff	7.32	3.77–10.88	59.4	*P* = 0.003

**Table 5:** Showing sub-group analysis of HCV prevalence by sample size, year of publication, and sub-regions of African countries

	Prevalence of HCV	95% confidence interval	Heterogeneity (I^2^%)	*P*-value
Sub-group analysis by sample size				
1. < 101	14.28	1.16–27.40	94.8	*P* = 0.001
2. 101–384	1.19	0.54–1.84	21.8	*P* = 0.269
3. 385–1000	7.04	2.46–11.62	97.6	*P* < 0.001
4. > 1000	1.26	0.67–1.85	-	-
Sub-group analysis by year of publication				
1. < 2011	3.74	2.24–5.23	94.2	*P* < 0.001
2. 2011–2021	17.94	17.94–24.75	95.7	*P* < 0.001
Sub-group analysis by regions of Africa				
1. North Africa	11.23	5.76–17.02	97.8	*P* < 0.001
2. East Africa	1.32	0.17–2.48	-	-
3. Middle Africa	1.69	0.004–3.33	-	-
4. Western Africa	3.04	1.08–4.99	65.5	*P* < 0.033
5. South Africa	2.90	1.04–4.78	-	-

The analysis of sub-group by sample size identified that highest prevalence of HBV among studies with sample size < 101, 11.33% (95% CI 6.17–16.50), and lowest prevalence was identified among sample size between 101 and 384, 5.57 (95% CI 4.31–6.83) (Table 4). Similarly, the highest prevalence of HCV among studies with sample size < 101, 14.28% (95% CI 1.16–27.40) and lowest prevalence was identified among sample size between 101 and 384, 1.19 (95% CI 0.54–1.84) (Table 5). The heterogeneity was shown to vary from 76.0 to 97.6% for HBV and from 21.8 to 97.6% for HCV.

In the sub-group analysis of studies by year of publication, highest prevalence of HBV was revealed by studies conducted before 2001, 12.33 (95% CI 3.32–21.39), and lowest among studies conducted between 2011 and 2021, 5.71 (95% CI 4.68–6.74) (Table 4). For HCV, lowest prevalence was revealed by studies conducted before 2001, 3.74 (95% CI 2.24–5.23), and highest among studies conducted between 2011 and 2021, 17.94 (95% CI 17.94–24.75) (Table 5).

The sub-group was also conducted by profession of HCWs for HBV, in which laboratory staffs were identified to have relatively highest prevalence, 7.32 (95% CI 3.77–10.88), and lowest prevalence were identified among physician 6.30 (95% CI 3.54–9.07). The heterogeneity was shown to vary from 59.7% among laboratory staff and to 84.4% among nurses (Table 4).

### Sensitivity analysis

To detect the source of heterogeneity, a leave-one-out sensitivity analysis was employed. The result of sensitivity analysis using the random-effects model revealed that there was no single study that influenced the overall prevalence of hepatitis B and C infection among HCWs (Supplementary file 3).

### The publication biases

**Fig. 4:**
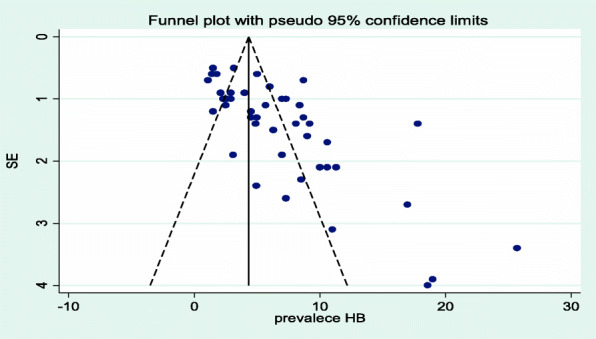
Funnel plot showing publication bias of studies included for the prevalence of hepatitis B among health care workers in Africa

The presence of publication bias was evaluated using funnel plots and Begg’s tests at a significance level of less than 0.05. The findings revealed that publication bias was significant for the studies on the prevalence of hepatitis B (*p* < 0.001) (Fig. 4). Similarly, it was statistically significant (*p* = 0.001) for the prevalence of hepatitis C among health care workers. The trim and fill analysis added twenty-nine studies and the pooled prevalence of hepatitis B in Africa varied to 9.1% (95% CI 7.1–11.7), while nine studies were added, and pooled prevalence of hepatitis C in Africa varied to 1.32 (95% CI 0.92–1.55).

### Meta-regression

In a meta-regression analysis, the publication year and sample size were not significant sources of heterogeneity for the prevalence of hepatitis B. In this study, no significant relationship was identified between the prevalence of hepatitis B and the publication year (*p*-value = 0.35) and sample size (*p*-value = 0.46). Likewise, there was no significant association between the prevalence of hepatitis C and the publication year (*p*-value = 0.67) and sample size (*p*-value = 0.84).

## Discussion

This is the first review and meta-analysis conducted in Africa on the prevalence of hepatitis B and C among HCWs. The pooled prevalence of hepatitis B and C among HCWs in Africa was 6.81% and 5.58%, respectively. The highest and lowest prevalence of HBV was identified in the western and northern parts of Africa, respectively.

The pooled prevalence of HBV among HCWs in Africa was not shown to have a significant difference from the general population (6.1%) [75]. The current finding was almost similar to a review conducted in Thailand which has revealed the pooled prevalence of hepatitis B among HCWs to be 5.2% [76]. The present prevalence of HBV identified by our analysis is higher than a review conducted in 2017 in Middle Eastern countries [77] and a study conducted in Turkey which has revealed hepatitis B prevalence among HCWs to be 3% [78]. It was also found to be much higher than the pooled prevalence of hepatitis B among HCWs reported in Iranians (0.4%) [79] and Brazilian 0.8% [80]. This may be due to the higher vaccination status of Iranians against hepatitis B [81] and the low vaccination status of African HCWs against hepatitis B [23]. Further, in developing countries, more than half of HBV infections in HCWs were attributable to percutaneous occupational exposure [21, 82].

The current review has revealed the prevalence of HCV among HCWs in Africa was nearly two times higher when compared with the prevalence in the general population (2.9%) [83]. This difference may be due to HCWs vulnerability towards occupational blood and body flood exposure than the general population. This finding is significantly higher when compared with the pooled prevalence reported in Germany (0.04%) [84], Turkey (0.3%) [78], and Scotland (0.28%) [85]. The possible reason for the discrepancy may be due to higher frequencies of healthcare associated infection in Africa than developed countries [86].

In this study, we found a variation in hepatitis B prevalence among HCWs across African regions. The sub-group analysis has revealed that the highest and lowest prevalence of hepatitis B among HCWs was found in the western and northern parts of Africa, respectively. The difference can be explained by the vaccination status of HCWs in the region. In the northern part of Africa, about 62% of HCWs have fully vaccinated against hepatitis B [23]. While only 30% of HCWs have been fully vaccinated in the western part of Africa [23]. The highest prevalence of HCV was identified in the northern part of Africa. The difference might be due to the higher occupational exposure rate of blood and body fluid in the northern part of Africa [21].

The strengths of this review and meta-analysis were comprehensive search through all reachable databases and rigorously following the PRISMA statement in all processes of conducting this systematic and meta-analysis.

This meta-analysis was reported with the following limitations. First, some studies included in this review had small sample sizes (*n* < 100 HCWs) which may affect the estimated prevalence of HBV and HCV in HCWs. As revealed by the sub-group analysis studies with a small sample size have shown higher prevalence of HBV (Table 4) and HCV (Table 5) among HCWs in Africa. This is supported by the fact that a small sample size will have a wider confidence interval and overestimate the magnitude of the association [87]. In addition, studies with a wide confidence interval are related to the low precision of the finding [88]. Second, there is a significant publication bias of included studies. Publication bias is bias caused by unpublished studies and usually researches with negative results less likely to be published [89]. Unpublished articles are not easily available in areas where there are no repositories. This is the most common problem in Africa since most research institutes and universities do not have repositories available online [90].

## Conclusion

The prevalence of hepatitis B is more than one in fifteen HCWs in Africa. While about one in twenty HCWs are affected by the hepatitis C virus. This high prevalence shows that hepatitis B and C are still endemic among HCWs in Africa. To reduce the prevalence of HCV and HBV among HCWs it needs a new strategy that reduces occupational exposure to blood and body fluids. Including mandatory vaccination against hepatitis B is required for HCWs as they are among the risky groups in the community and reduction of occupational exposure by maintaining adequate personal protective equipment supported by regulations. In addition, continuous training on infection prevention procedures for all HCWs should be provided.

## Supplementary Information

**Additional file 1.** PRISMA 2009 Checklist.

**Additional file 2.** Searched data base with search strategy.

**Additional file 3: Table 1.** Sensitivity analysis of prevalence of HBsAG among health care workers in Africa for each study being removed at a time, 1989-2020. **Table 2.** Sensitivity analysis of prevalence of anti – HCV among health care workers in Africa for each study being removed at a time, 1989-2020.

## Data Availability

The part of the data analyzed during this study is included in this manuscript. Other data will be available from the corresponding author upon a reasonable request.
