# Supplementary material for: Hepatitis B and C virus infection among healthcare workers in Africa: a systematic review and meta-analysis
Source: Environ Health Prev Med. 2021 Jun 2;26:61. doi: 10.1186/s12199-021-00983-9 (PMC8173813; doi:10.1186/s12199-021-00983-9)
Supplement: Supplementary file 2 — Additional file 2. Searched data base with search strategy. [file 12199_2021_983_MOESM2_ESM.docx]

Searched data base with search strategy

**PubMed**

Search: **(((prevalence) OR (magnitude)) AND (("hepatitis B") OR ("hepatitis C")) AND infection AND (("health care workers") OR ("medical personnel")) AND (Africa [mesh]))**

("epidemiology"[MeSH Subheading] OR "epidemiology"[All Fields] OR "prevalence"[All Fields] OR "prevalence"[MeSH Terms] OR "prevalance"[All Fields] OR "prevalences"[All Fields] OR "prevalence s"[All Fields] OR "prevalent"[All Fields] OR "prevalently"[All Fields] OR "prevalents"[All Fields] OR ("magnitude"[All Fields] OR "magnitudes"[All Fields])) AND ("hepatitis B"[All Fields] OR "hepatitis C"[All Fields]) AND ("infect"[All Fields] OR "infectability"[All Fields] OR "infectable"[All Fields] OR "infectant"[All Fields] OR "infectants"[All Fields] OR "infected"[All Fields] OR "infecteds"[All Fields] OR "infectibility"[All Fields] OR "infectible"[All Fields] OR "infecting"[All Fields] OR "infection s"[All Fields] OR "infections"[MeSH Terms] OR "infections"[All Fields] OR "infection"[All Fields] OR "infective"[All Fields] OR "infectiveness"[All Fields] OR "infectives"[All Fields] OR "infectivities"[All Fields] OR "infects"[All Fields] OR "pathogenicity"[MeSH Subheading] OR "pathogenicity"[All Fields] OR "infectivity"[All Fields]) AND ("health care workers"[All Fields] OR "medical personnel"[All Fields]) AND "africa"[MeSH Terms]

**Translations**

**Prevalence:** "epidemiology"[Subheading] OR "epidemiology"[All Fields] OR "prevalence"[All Fields] OR "prevalence"[MeSH Terms] OR "prevalance"[All Fields] OR "prevalences"[All Fields] OR "prevalence's"[All Fields] OR "prevalent"[All Fields] OR "prevalently"[All Fields] OR "prevalents"[All Fields]

**Magnitude:** "magnitude"[All Fields] OR "magnitudes"[All Fields]

**infection:** "infect"[All Fields] OR "infectability"[All Fields] OR "infectable"[All Fields] OR "infectant"[All Fields] OR "infectants"[All Fields] OR "infected"[All Fields] OR "infecteds"[All Fields] OR "infectibility"[All Fields] OR "infectible"[All Fields] OR "infecting"[All Fields] OR "infection's"[All Fields] OR "infections"[MeSH Terms] OR "infections"[All Fields] OR "infection"[All Fields] OR "infective"[All Fields] OR "infectiveness"[All Fields] OR "infectives"[All Fields] OR "infectivities"[All Fields] OR "infects"[All Fields] OR "pathogenicity"[Subheading] OR "pathogenicity"[All Fields] OR "infectivity"[All Fields]

**Africa [mesh]:** "africa"[MeSH Terms]

# **Cochrane library**

Cochrane Reviews matching **"prevalence" in Title Abstract Keyword AND "hepatitis-B" in Title Abstract Keyword OR "hepatitis C" in Title Abstract Keyword AND "health care workers" in Title Abstract Keyword AND "Africa" in Title Abstract Keyword**

# **Popline**

((magnitude) OR (prevalence)) AND (hepatitis B) AND (hepatitis C) AND (Infection) AND (("health care workers") OR ("medical personnel")) AND (Africa)

**AJOL**

(Magnitude OR prevalence) AND ("Hepatitis B" OR "Hepatitis C") AND "health care workers" AND Africa

**Henari**

(Title Combined :( Hepatitis B)) AND (Title Combined :( Hepatitis C)) AND ("Health Care Workers") AND (Africa)

**CINAL Through Queens University**

**(Prevalence) AND (hepatitis B and hepatitis C) AND health care workers AND Africa**

**Science direct**

((magnitude) OR (prevalence)) AND (hepatitis B) AND (hepatitis C) AND (Infection) AND (("health care workers") OR ("medical personnel")) AND (Africa).
