# Supplementary material for: Hepatitis B and C virus infection among healthcare workers in Africa: a systematic review and meta-analysis
Source: Environ Health Prev Med. 2021 Jun 2;26:61. doi: 10.1186/s12199-021-00983-9 (PMC8173813; doi:10.1186/s12199-021-00983-9)
Supplement: Supplementary file 3 — Additional file 3: Table 1. Sensitivity analysis of prevalence of HBsAG among health care workers in Africa for each study being removed at a time, 1989-2020. Table 2. Sensitivity analysis of prevalence of anti – HCV among health care workers in Africa for each study being removed at a time, 1989-2020. [file 12199_2021_983_MOESM3_ESM.docx]

Table 1: Sensitivity analysis of prevalence of HBsAG among health care workers in Africa for each study being removed at a time, 1989-2020.

| Name , year | Pooled prevalence | 95% CI | I^2^ (%) | Q | p-value |
| --- | --- | --- | --- | --- | --- |
| Desalegn et al , 2013 | 6.94 | 5.78 – 8.11 | 91.8 | 473.73 | < 0.001 |
| Ziraba et al , 2010 | 6.78 | 5.62 – 7.92 | 91.7 | 470.04 | < 0.001 |
| Mueller et al, 2016 | 6.81 | 5.65 – 7.92 | 91.1 | 469.91 | < 0.001 |
| Nail et al, 2008 | 6.38 | 5.31 – 7. 45 | 91.0 | 476.02 | < 0.001 |
| Abdewaheb et al, 2012 | 6.91 | 5.84 – 8.12 | 91.3 | 445.24 | < 0.001 |
| Braka et al ,2006 | 6.71 | 5.85 – 7.76 | 91.9 | 471.78 | < 0.001 |
| Djeriri et al ,2008 | 6.83 | 5.74 – 7.92 | 91.0 | 479.07 | < 0.001 |
| Ngekegn et al, 2018 | 6.81 | 5.74 – 7.87 | 91.0 | 480.14 | < 0.001 |
| Elmaghloub et al, 2017 | 6.91 | 5.84 – 7.97 | 91.5 | 455.01 | < 0.001 |
| Elmukshafi et al, 2012 | 6.79 | 5.71 – 7.86 | 91.0 | 475.85 | < 0.001 |
| Elduma et al, 2006 | 6.81 | 5.74 – 7.87 | 91.0 | 480.23 | < 0.001 |
| Fritzsche et al, 2015 | 6.77 | 5.71 – 7.84 | 91.0 | 478.65 | < 0.001 |
| Gebremariam et al ,2018 | 6.82 | 5.75 – 7.89 | 91.0 | 480.37 | < 0.001 |
| Munir et al, 2013 | 6.75 | 5.69 – 7.81 | 91.9 | 471.34 | < 0.001 |
| Kisangau et al, 2018 | 6.82 | 5.75 – 7.89 | 91.0 | 480.38 | < 0.001 |
| Jean-Baptiste et al, 2018 | 6.71 | 5.66 – 7.87 | 90.8 | 466.41 | < 0.001 |
| Souly et al, 2016 | 6.89 | 5.79 – 7.99 | 91.9 | 474.42 | < 0.001 |
| Orji et al, 2020 | 6.89 | 5.82 – 7.95 | 91.9 | 474.11 | < 0.001 |
| Yezengaw et al, 2018 | 6.87 | 5.80 – 7.94 | 91.0 | 477.36 | < 0.001 |
| Ndako et al, 2014 | 6.58 | 5.54 – 7.61 | 90.6 | 458.29 | < 0.001 |
| Elikwu et al, 2016 | 6.76 | 5.70 – 7.82 | 91.0 | 478.40 | < 0.001 |
| Geberemichael et al, 2013 | 6.75 | 5.70 – 7.81 | 91.0 | 479.09 | < 0.001 |
| Shoa et al , 2018 | 6.79 | 5.73 – 7.86 | 91.0 | 478.81 | < 0.001 |
| Saldone et al ,2016 | 6.87 | 5.80 – 7.94 | 91.0 | 478.32 | < 0.001 |
| Tastilong et al, 2016 | 6.70 | 5.70 – 7.75 | 91.0 | 475.75 | < 0.001 |
| Kateera et al, 2014 | 6.87 | 5.80 – 7.94 | 91.0 | 477.82 | < 0.001 |
| Akazong et al, 2020 | 6.72 | 5.77 – 7.95 | 90.8 | 466.66 | < 0.001 |
| Amiwero et al, 2017 | 6.56 | 5.72 – 7.92 | 90.8 | 469.31 | < 0.001 |
| Daw et al, 2000 | 6.84 | 5.80 – 7.92 | 91.0 | 480.27 | < 0.001 |
| Ramieu et al ,1989 | 6.40 | 5.43 – 7.26 | 89.9 | 386.65 | < 0.001 |
| Yu-Lig- Qin et al, 2018 | 6.59 | 5.64 – 7.75 | 90.8 | 473.06 | < 0.001 |
| Elsouki et al, 2014 | 6.40 | 5.33 – 7.47 | 90.7 | 461.48 | < 0.001 |
| Ndogo et al, 2016 | 6.68 | 5.65 – 7.72 | 90.2 | 439.40 | < 0.001 |
| Masaka et al, 2018 | 6.63 | 5.59 – 7.67 | 90.8 | 467.64 | < 0.001 |
| Massaqui et al , 2018 | 6.71 | 5.65 – 7.76 | 90.8 | 468.92 | < 0 .001 |
| Mbaawaga et al, 2019 | 6.68 | 5.63 – 7.73 | 90.9 | 471.42 | < 0.001 |
| Sani et al, 2011 | 6.72 | 5.57 – 7.66 | 90.8 | 466.32 | < 0.001 |
| Zayet et al, 2015 | 6.84 | 5.78 – 7.90 | 91.9 | 479.98 | < 0.001 |
| Kefenie et al,201 | 6.70 | 5.14 – 7.25 | 90.8 | 468.13 | < 0.001 |
| Belo et al,2000 | 6.48 | 5.48 – 7.50 | 90.2 | 440.80 | < 0.001 |
| Gyang et al. 2016 | 6.27 | 5.72 – 7.82 | 90.8 | 480.4 | < 0.001 |

Table 2: Sensitivity analysis of prevalence of anti – HCV among health care workers in Africa for each study being removed at a time, 1989-2020.

| Name , year | Pooled prevalence | 95% CI | I^2^ (%) | Q | p-value |
| --- | --- | --- | --- | --- | --- |
| Abdewaheb et al. 2012 | 4.13 | 2.59 – 5.67 | 90.7 | 118.35 | < 0.001 |
| Fritzsche et al. 2015 | 6.01 | 3.81 – 8.20 | 95.5 | 243.97 | < 0.001 |
| Munir et al. 2013 | 5.41 | 3.32 – 7.49 | 95.1 | 223.58 | < 0.001 |
| Jean-Baptiste et al. 2018 | 6.17 | 3.79 – 8.55 | 95.4 | 239.37 | < 0.001 |
| Souly et al. 2016 | 6.25 | 3.77 – 8.73 | 95.1 | 223.60 | < 0.001 |
| Kateera et al. 2014 | 6.11 | 3.82 – 8.39 | 95.4 | 240.49 | < 0.001 |
| Elbahrawy et al. 2017 | 5.25 | 3.21 – 7.29 | 94.9 | 216.46 | < 0.001 |
| Amiwero et al. 2017 | 5.92 | 3.73 – 8.11 | 95.5 | 244.76 | < 0.001 |
| Vardas et al. 2002 | 6.05 | 3.79 – 8.38 | 95.5 | 243.82 | < 0.001 |
| Sani et al. 2011 | 5.63 | 3.52 – 7.73 | 95.5 | 243.34 | < 0.001 |
| Zayet et al. 2015 | 5.58 | 3.55 – 7.61 | 95.1 | 244.76 | < 0.001 |
| El-Sokkary et al. 2017 | 4.77 | 2.95 – 6.56 | 94.2 | 206. 49 | < 0.001 |
| Gyang et al. 2016 | 5.48 | 3.47 – 7.49 | 95.1 | 243. 91 | < 0.001 |
